# A sesquiterpene lactone, tomentosin, as a novel anticancer agent: orchestrating apoptosis, autophagy, and ER stress in colorectal cancer

**DOI:** 10.1007/s00210-025-04111-0

**Published:** 2025-04-11

**Authors:** Sümeyra Çetinkaya, Ebru Güçlü, İlknur Çınar Ayan, Hasibe Vural, Hatice Gül Dursun

**Affiliations:** 1 Biotechnology Research Center, Field Crops Central Research Institute, Ankara, 06170 Türkiye; 2 Department of Basic Science and Health, Hemp Research Institute, Yozgat Bozok University, Yozgat, Türkiye; 3 Department of Medical Biology, Faculty of Medicine, Necmettin Erbakan University, Konya, Türkiye

**Keywords:** Tomentosin, Colorectal cancer, ER stress, Apoptosis, Autophagy

## Abstract

Colorectal cancer (CRC) remains a leading cause of cancer-related mortality worldwide. Natural compounds with anticancer potential, such as tomentosin, a sesquiterpene lactone derived from *Inula viscosa*, are under investigation as alternative therapeutic agents. However, its potential effects on CRC remain unexplored. This study aimed to evaluate the anticancer potential of tomentosin in CRC cells and elucidate its underlying molecular mechanisms. HCT 116 and HT- 29 cells were treated with tomentosin, and its effects on cell viability, colony formation, invasion, apoptosis, mitochondrial membrane potential (MMP), reactive oxygen species (ROS) production, autophagy, and endoplasmic reticulum (ER) stress were evaluated. Various assays, including XTT, colony formation, and Matrigel invasion assays, were used to assess cell viability, proliferation, and invasion. Tomentosin markedly reduced cell viability and colony formation in a dose-dependent manner. It suppressed invasion and induced apoptosis, as evidenced by an increased apoptotic index and upregulation of *CASP3*, *CASP7*, *CASP8*, *CASP9*, and *BAX*. Tomentosin disrupted MMP and elevated ROS levels, contributing to apoptotic signaling. Autophagic activity was significantly upregulated, with increased expression of *BECLIN1*, *ATG5*, *ATG7*, and *MAP1LC3 A*. ER stress markers *GRP78*, *ATF6*, *CHOP*, and *XBP1* were also upregulated, suggesting a role in cell death. Tomentosin has anticancer effects in CRC cells by inducing apoptosis, modulating autophagy, and triggering ER stress. These findings underscore tomentosin’s potential as a novel therapeutic candidate for CRC, warranting further in vivo and clinical investigations.

## Introduction

Colorectal cancer (CRC) is a malignant adenocarcinoma, which develops primarily in the colon and rectum. It is a high prevalence disease with high mortality rates creating issues in both health and economy by globally affecting both genders. It is the second most prevalent cause of cancer-related deaths globally and is estimated that approximately 1.9 million new cases occur each year. Although the 5-year survival rate is over 90% for localized stages, this rate drops to approximately 70% in locally advanced stages and is known to be less than 20% in metastatic ones (Arnold et al. 2017; Klimeck et al. 2023; Bray et al. 2024). At all stages of this cancer, women tend to survive at higher rates compared to men, and younger patients generally have higher survival rates compared to older ones. Because CRC has a high tendency to metastasize, it usually spreads to distant organs of the body such as the liver, lungs, and lymph nodes (Eschrich et al. 2005; Ahmed et al. 2013; Yang et al. 2017). Therefore, early diagnosis and effective treatment approaches are crucial, as they can significantly affect the prognosis of the disease.

Current treatment options for CRC include surgery, chemotherapy (such as 5-fluorouracil (5-FU), capecitabine, oxaliplatin), radiotherapy, immunotherapy (such as pembrolizumab, nivolumab), and targeted therapies (such as bevacizumab, cetuximab, panitumumab) (Ciardiello et al. 2022; Wong et al. 2023). Among these options, chemotherapy drugs effectively kill cancer cells but are known for their serious side effects. In addition to these side effects, the development of resistance to chemotherapy remains one of the biggest challenges in treatment. In this regard, researching alternative treatment options with fewer side effects continues to be a subject of great interest (Mokhtari et al. 2017).

Because of their numerous bioactive effects, capacity of appropriateness for combination treatments, low adverse reactions, targeting multiple biological pathways, and capacity to slow the development of resistance, natural compounds derived from plants are being extensively analyzed through studies in the cancer research field (Asma et al. 2022). The sesquiterpene lactone tomentosin, which was discovered from *Inula* species within the Asteraceae family, is noteworthy among them due to its many biological activities and pharmacological properties (Mamoci et al. 2011; Aydin et al. 2022). Some investigations have different claims including tomentosin has anti-inflammatory (Eli Omari et al. 2021), antioxidant (Yang et al. 2022), and anticancer (Lee et al. 2019) qualities. In addition, it has antitumor effects in some cancers, including melanoma (Rozenblat et al. 2008), breast (Li et al. 2011), osteosarcoma (Lee et al. 2019), gastric (Yang et al. 2020), neuroblastoma (He et al. 2020), hepatocellular (Yu et al. 2021), leukemia (Yang et al. 2021), and pancreatic (Güçlü et al. 2022).

Due to its pro-apoptotic and stress-regulating qualities in numerous cancer types, tomentosin is one of the promising therapeutic agents for cancer therapy. To the best of our knowledge, there is no studies specifically investigating the effect of tomentosin on colorectal cancer, which creates a notable gap in the literature. To address this, the present study explores the anticancer activity of tomentosin in the CRC cell lines HCT 116 and HT- 29 at both cellular and molecular levels, with a focus on whether tomentosin induces apoptosis via mitochondrial dysfunction, triggers autophagy as either a survival or death mechanism, modulates endoplasmic reticulum (ER) stress, and contributes to oxidative stress regulation.

## Material and methods

### Cell culture and treatment

HT- 29 (*HTB- 38*™) and HCT 116 (*CCL- 247*™) cell lines were obtained from the American Type Culture Collection (ATCC, Manassas, VA, USA). These cell lines were selected due to their differences in chemotherapy resistance and distinct genetic mutations. This selection was made to comprehensively evaluate the effects of tomentosin on various biological mechanisms. Cells were cultured in high glucose DMEM (DMEM-HA, Capricorn Scientific) enriched with 10% fetal bovine serum (FBS) (FBS-HI- 11 A, Capricorn Scientific) and 1% penicillin–streptomycin (100 U/mL- 10 mg/mL) (PS-B, Capricorn Scientific). The cultures were maintained in a humidified incubator at 37 °C with 5% CO_2_ and 95% humidity. Tomentosin (HY-N8284, MedChemExpress) was dissolved in dimethyl sulfoxide (DMSO) (Sigma-Aldrich) to prepare a stock solution, which was diluted to the required concentrations for experiments and stored at − 20 °C until used.

### Cell viability

The cytotoxic effect of tomentosin on HT- 29 and HCT 116 cells was evaluated using the XTT assay (Cat# 20–300–1000, Biological Industries). Cells were seeded into 96-well plates at a density of 1 × 10^4^ cells per well and incubated for 24 h. Following incubation, cells were treated with different doses of tomentosin (2.5, 5, 10, 20, 50 µM) for 24, 48, and 72 h, ensuring that the final DMSO concentration did not exceed 0.1% (v/v). After the treatment period, 100 µL of XTT reaction solution was added to each well, and the plates were incubated for an additional 4 h at 37 °C. Absorbance measurements were performed using an ELISA plate reader at 450 nm. Cell viability for each condition was calculated as a percentage relative to the control group (Çınar Ayan et al. 2022).

### Colony assay

A colony formation assay was performed to understand the long-term effects of tomentosin on the proliferative capacity of HT- 29 and HCT 116 cells. Both cell types were seeded into six-well plates at a density of 2 × 10^3^ cells per well. After allowing the cells to adhere for 24 h at 37 °C, they were treated with tomentosin. To maintain optimal growth conditions, the culture medium was refreshed every 2 days. At the end of the 12-day incubation period, the cells were gently washed with PBS, then fixed with 100% methanol at − 20 °C for 10 min. Colonies were visualized by staining with 1% crystal violet solution for 15 min. Images of the colonies were captured, and the number of colonies in the treated and control groups was counted under a microscope (Çınar Ayan et al. 2022).

### Cell death detection assay

To assess histone-associated DNA fragments, the Cell Death Detection ELISA^plus^ kit (Cat#11,774,425,001, Sigma Aldrich) was used following the manufacturer’s instructions. HT- 29 and HCT 116 cells were seeded into 96-well plates at a density of 1 × 10^4^ cells per well. After incubation, the cells were treated with tomentosin. To obtain cytoplasmic lysates, cells were centrifuged at 200 × g for 10 min, and the resulting cell pellet was resuspended in 200 µL of lysis buffer for each well. The cells were then incubated at 25 °C for 30 min. The cell lysates were transferred into streptavidin-coated microplates, with 20 µL added to each well. The samples were then treated incubated with an anti-histone-biotin antibody. To determine the presence of nucleosomes, unbound substances were removed by washing, and 2,20-azino-di-(3-ethylbenzthiazoline sulfonate) (ABTS) substrate was added. Absorbance was measured at 405 nm using an ELISA plate reader.

### Cell invasion

To evaluate the potential effects of tomentosin on cell invasion, an invasion assay was performed using the Corning® BioCoat™ Matrigel® Invasion Chamber (Cat#354,480, Corning Inc.). This assay is an essential method to determine whether tomentosin inhibits the invasive behavior of the cancer cells. For this experiment, cell suspensions were prepared for both the control and tomentosin-treated groups at a density of 2.5 × 10^5^ cells/mL in serum-free media. Then, 500 µL of the cell suspension containing equal number of cells was added to the upper chambers, while the lower chambers was filled with serum-enriched media as a chemoattractant. After 22 h, cells that migrated to the lower side of the chamber were fixed with 100% methanol and stained with 0.1% crystal violet. To assess the regulatory effects of tomentosin on cell invasion, the number of invading cells was counted under a light microscope (Güçlü et al. 2022).

### Grp78/Bip ELISA assay

To investigate the effects of tomentosin on cellular stress pathways, the Enzo GRP78/BiP ELISA kit (Cat#ADI- 900–214, Enzo Life Sciences) was used to measure GRP78/BiP protein levels. The assay was performed according to the manufacturer’s instructions. First, cell pellets were prepared and treated with 1X extraction reagent (containing protease inhibitor cocktail and phenylmethylsulfonyl fluoride (PMSF)), followed by incubation on ice for 30 min. The centrifuged cell lysates were stored at − 20 °C until use. During the assay, 100 µL of assay buffer and standard solutions were added to each well of the 96-well plates. Then, 100 µL of cell lysates were pipetted into each well, followed by the addition of 50 µL of yellow antibody solution and 50 µL of blue conjugate, the plate was then incubated. After washing with 1 × wash buffer, 200 µL of TMB solution was added to each well and incubated for 30 min. The reaction was terminated by adding 50 µL of stop solution. Absorbance values ​​were measured with an ELISA reader at a wavelength of 450 nm.

### Intracellular reactive oxygen species (ROS) detection

To understand how tomentosin alters the oxidative balance in cells, intracellular ROS levels were measured using ROS Red Dye (Cat#ab186027, Abcam). After the cells reached sufficient confluency, 100 μL of ROS red working solution was added to both the control and tomentosin-treated cells. The cells were then incubated at 37 °C for 1 h. After incubation, 20 μL of assay buffer was added to each well, and following 15 min later, red fluorescence was measured using a fluorescence microplate reader (BioTek Epoch) at a wavelength of 520 nm (Güçlü et al. 2022).

### Assessment of mitochondrial membrane potential (MMP)

As a critical indicator of apoptosis, the effect of tomentosin on MMP was evaluated to determine whether it triggers mitochondrial dysfunction. For this, the TMRM assay (ab228569, Abcam) was performed according to the manufacturer’s instructions. Briefly, TMRM working solution was added to both tomentosin-treated and untreated cells, and the cells were incubated in the dark at 37 °C for 45 min. At the end of the incubation period, the cells were aspirated and 1 × live cell imaging buffer was added. Changes in MMP were visualized using a fluorescence microscope (Ex/Em = 548/573). The fluorescence intensity was quantified to assess the relative differences in MMP between treated and untreated groups.

### RNA extraction and real-time quantitative PCR (RT-qPCR) analysis

To elucidate the molecular mechanisms underlying tomentosin’s effects on colorectal cancer cells, the mRNA expression levels of genes involved in apoptosis (*CASP3, CASP7, CASP8, CASP9, BAX, BCL2, CYCS, TNFA, TNFR1, FADD*), autophagy (*ATG5, ATG7, ATG12, MAP1LC3 A, MAP1LC3B*), and ER stress *(ERN1, PERK, ATF6, GRP78, CHOP, XBP1, ATF4, ASK1, GADD34, TRAF2, EIF2 A*) were assessed using RT-qPCR. The genes were selected based on their well-established roles in apoptotic, autophagic, and ER stress pathways via literature reviews from databases such as PubMed (https://pubmed.ncbi.nlm.nih.gov). This comprehensive gene expression analysis was conducted to gain insights into how tomentosin modulates these cellular pathways, potentially contributing to its anticancer effects in colorectal cancer. RNA isolation (301–001, GeneAll) and cDNA synthesis (170–8891, Bio-Rad) were performed according to the manufacturer's instructions. RT-qPCR analyses were conducted using BrightGreen 2X qPCR MasterMix (Brightgreen MasterMix-R, abm) on a Bio-Rad CFX Connect™ Real-Time System. Primers for the target genes and reference genes were designed using IDT PrimerQuest (https://eu.idtdna.com/Primerquest/Home/Index).

### Autophagy detection

The CYTO-ID® Autophagy Detection Kit (ENZ- 51031–0050, Enzo Life Sciences), which enables qualitative and quantitative detection of autophagic vacuoles in live cells, was used to investigate the effect of tomentosin on autophagy in CRC cells. The kit was employed according to the manufacturer’s instructions. Briefly, CRC cells were seeded at a density of 1 × 10⁶ cells/mL. Once the cells reached confluency, they were washed with assay buffer. Following this step, the cells were incubated with the CYTO-ID Green Detection Reagent at 37 °C in the dark. A fluorescence microscope was used to examine stained cells that contained autophagic vacuoles.

### Statistical analysis

Each experiment was conducted independently three times, and the results are expressed as the mean ± standard deviation (SD). Statistical comparisons between groups were performed using Student's *t*-test with GraphPad Prism software (version 10.0.2; GraphPad Software, Inc., La Jolla, CA, USA). Additionally, variations in mRNA expression levels among groups were analyzed using the RT^2^ Profiler™ PCR Array Data Analysis, an advanced online analytical tool designed for gene expression studies. A *p*-value below 0.05 was considered statistically significant in all analyses.

## Results

### Tomentosin decreased the cell viability and colony formation of colorectal cancer cells

**Fig. 1:**
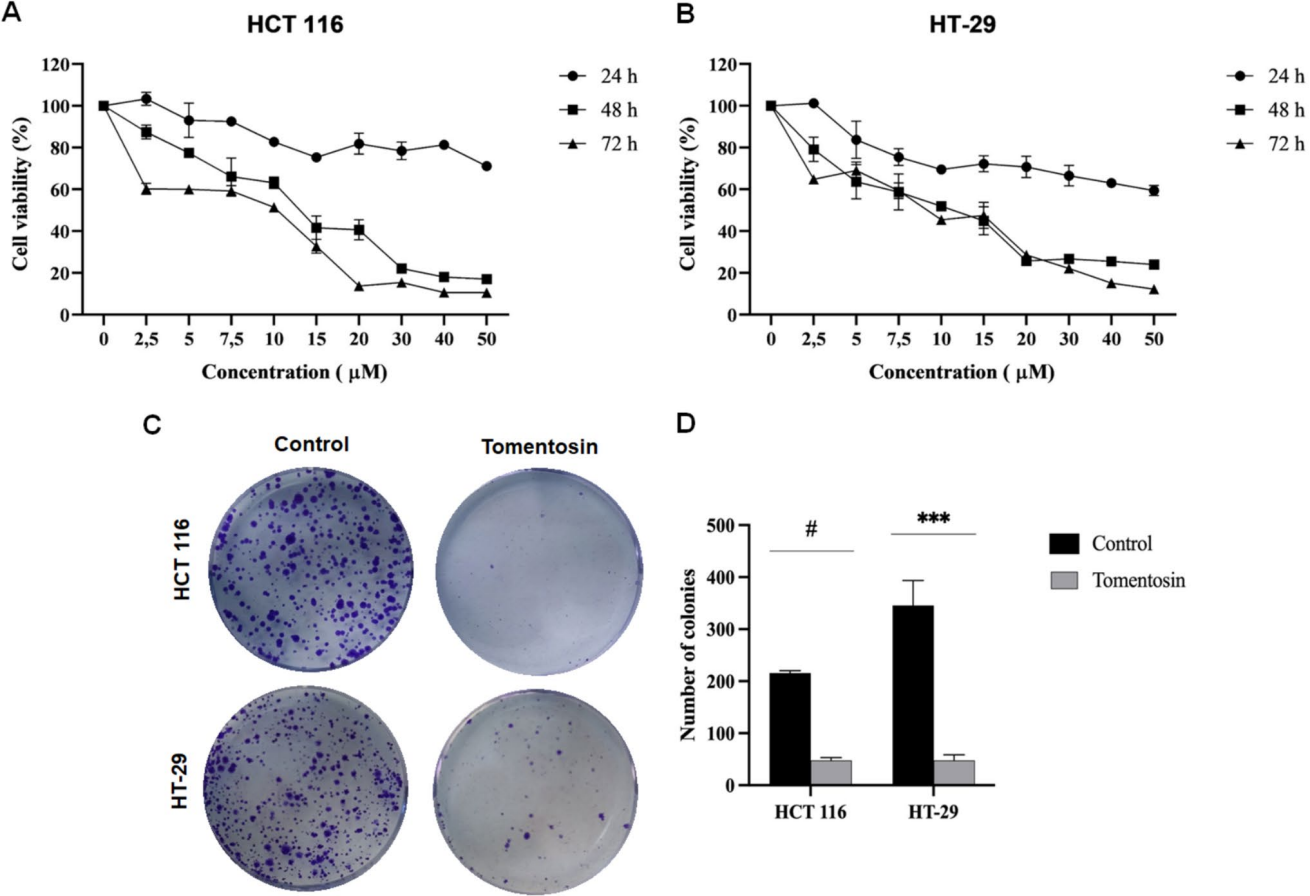
Dose- and time-dependent effects of tomentosin on cell viability of HCT 116 (**A**) and HT- 29 (**B**) cells, as measured by XTT assay. **C** Representative images of colony formation assay showing the long-term effects of tomentosin. Control groups had significantly more colonies compared to the tomentosin-treated groups. **D** Quantification of colony numbers in both cells following tomentosin treatment. Colony formation was significantly reduced in both cell lines compared to the control group (^***^p < 0.001, ^#^*p* < 0.0001)

The XTT assay was performed at 24, 48, and 72-h time periods with various concentrations of tomentosin (2.5–50 µM) in HCT 116 and HT- 29 cells. Cell viability was inhibited by tomentosin treatment in a dose-and time dependent manner. IC_50_ doses ​​of tomentosin in HCT 116 and HT- 29 cells for 48 h were determined as 13.30 ± 1.20 and 10.01 ± 1.56, respectively (Fig. 1A and B). IC_50_ doses of tomentosin in HCT 116 and HT- 29 cells for 72 h were calculated as 8.51 ± 0.67 and 9.91 ± 1.37 µM, respectively. In 24-h tomentosin treatment, 50% viability could not be achieved even at the highest dose. Considering the % viability values, it was decided that 48 h was more appropriate for both lower dose and relatively shorter tomentosin treatment. In other analyses of this study, cells were treated with IC_50_ doses of tomentosin for 48 h. Accordingly, colony formation was significantly suppressed in tomentosin-treated cells compared to the control group, indicating that tomentosin inhibits colonogenic survival (Fig. 1C). In HCT 116 cell lines, the number of colonies was 210 ± 15 in the control group and 45 ± 5 in the tomentosin-treated group (*p* < 0.0001). Similarly, in HT- 29 cells, colony formation was decreased from 425 ± 20 in the control group to 60 ± 10 in the tomentosin-treated group (*p* < 0.001) (Fig. 1D).

### Tomentosin reduced invasion, triggers apoptosis, and affects the expression levels of GRP78/BiP in HCT 116 and HT- 29 cells

**Fig. 2:**
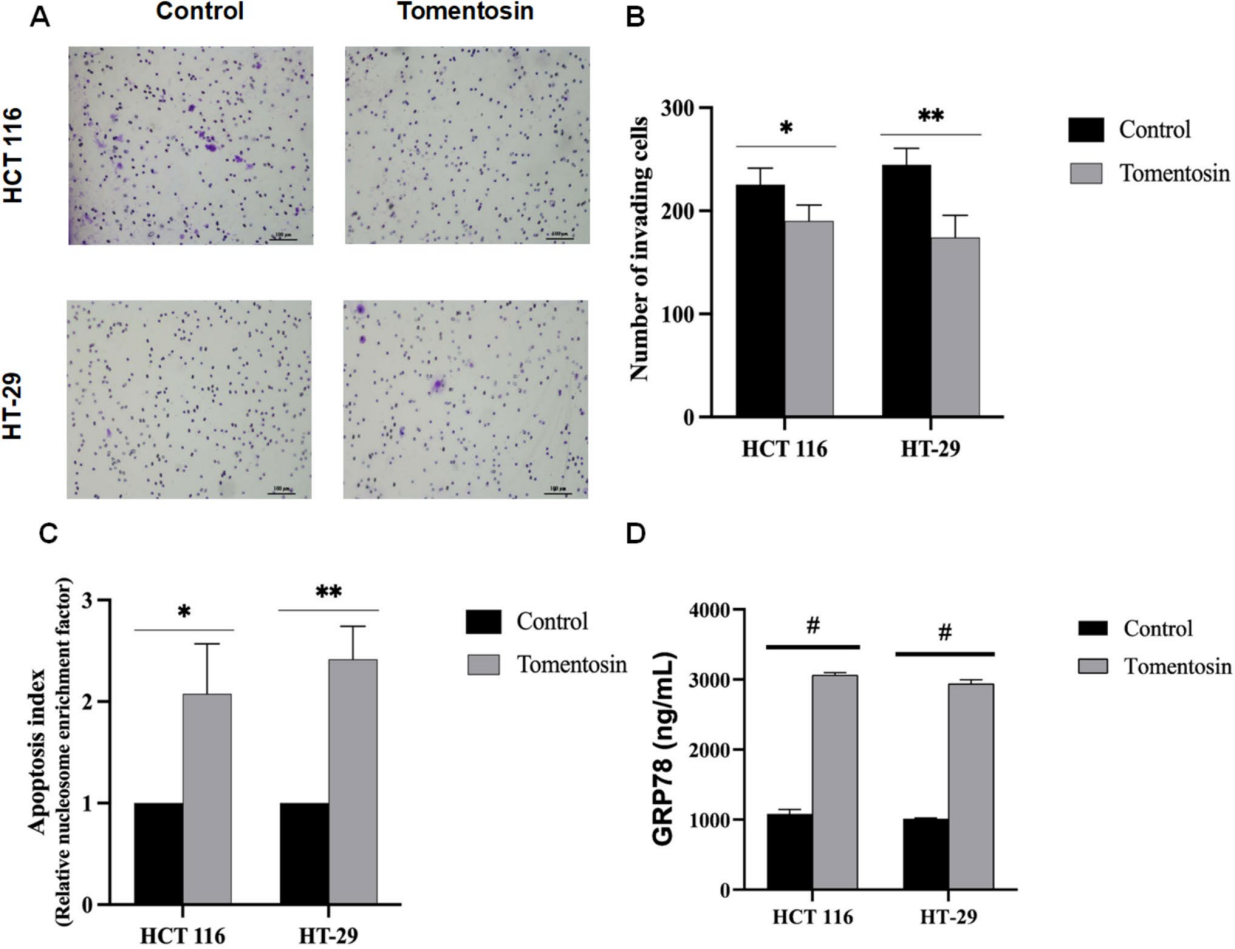
Images representing cell invasion after tomentosin treatment in colorectal cancer cells. Crystal violet staining highlights invaded cells (**A**, **B**). Apoptosis index as determined by the Cell Death Detection ELISA assay. Tomentosin treatment led to a 2.5-fold increase in HCT 116 cells and a 2.8-fold increase in HT- 29 cells compared to controls (**C**). GRP78/BiP protein levels were significantly elevated in tomentosin-treated cells compared to controls in both cell lines (**D**) (^*^p < 0.05, ^**^p < 0.01, ^#^*p* < 0.0001)

Tomentosin treatment significantly reduced the number of invading HCT 116 cells by 20%, from 220 in the control group to 175 (*p* < 0.05). In HT- 29 cells, the number of invading cells decreased from 250 to 170, demonstrating a significant anti-invasive effect (*p* < 0.01) (Fig. 2A and B). In addition to its effect on cell invasion, tomentosin treatment significantly increased apoptosis in both cell lines, as evidenced by the increased apoptosis index (Fig. 2C). Compared to the control group, tomentosin treatment resulted in a 2.5-fold increase in the apoptosis index in HCT 116 cells (*p* < 0.05) and a 2.8-fold increase in HT- 29 cells (*p* < 0.01), as shown in Fig. 2C. GRP78/BiP protein levels, one of the key markers of endoplasmic reticulum (ER) stress, showed significantly higher protein levels in tomentosin-treated cells compared to controls in both HCT 116 and HT- 29 cells (*p* < 0.0001) (Fig. 2D).

### Tomentosin disrupted MMP, increases intracellular ROS levels in HCT 116 and HT- 29 cells

**Fig. 3:**
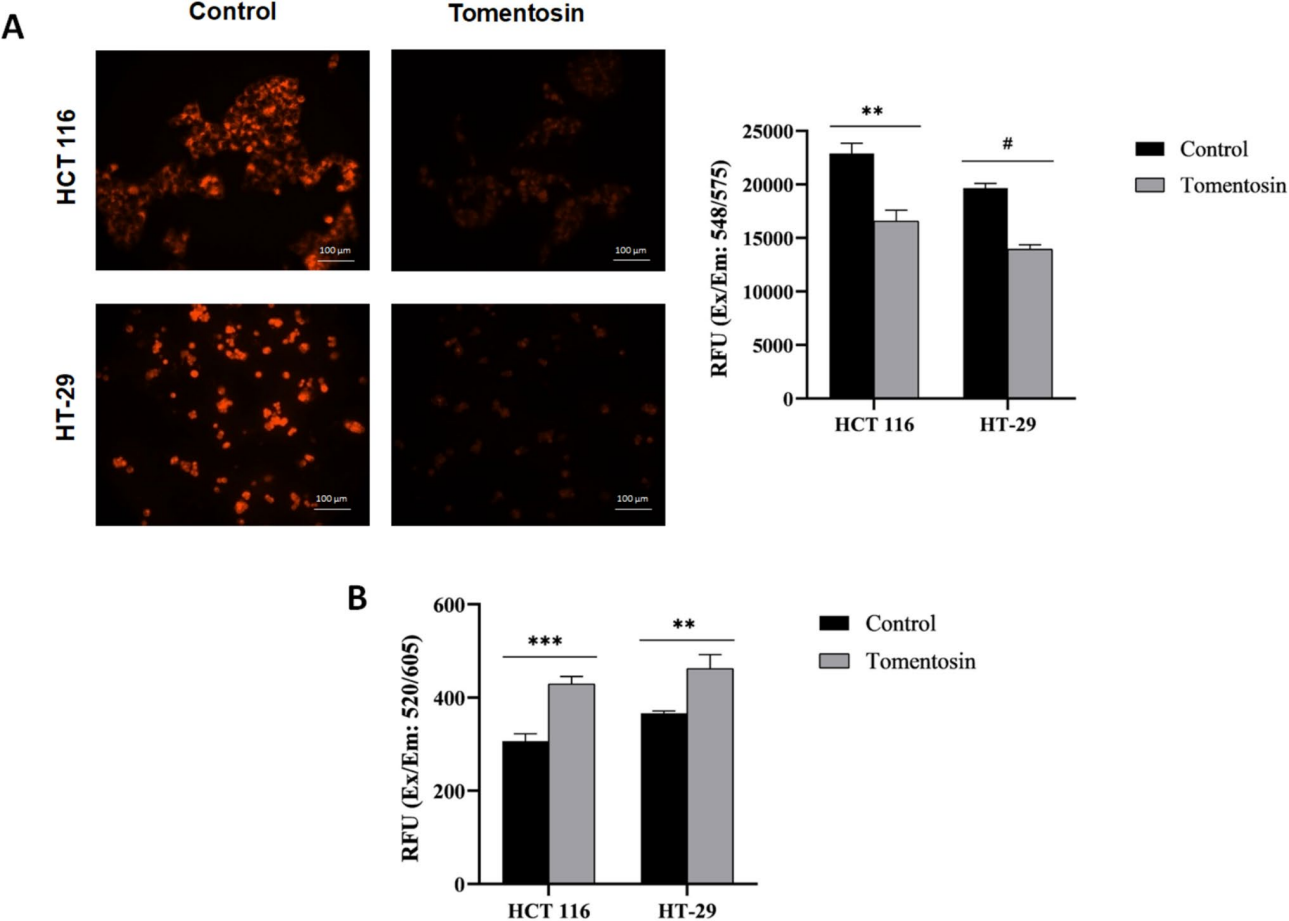
Tomentosin disrupted MMP and increases intracellular ROS levels in HCT 116 and HT- 29 cells. Representative fluorescence microscopy images showing MMP loss in HCT 116 and HT- 29 cells following tomentosin treatment. MMP changes and quantitative analysis, based on the TMRM assay (Ex/Em: 548/575 nm), revealed a significant decrease in MMP in HCT 116 and HT- 29 cells after tomentosin treatment (**A**). Similarly, the DCFDA assay (Ex/Em: 520/605 nm) showed a significant increase in intracellular ROS levels in both cell lines. RFU: Relative Fluorescence Units (**B**) (^**^p < 0.01, ^***^p < 0.001, ^#^*p* < 0.0001)

The TMRM assay revealed a significantly decrease in MMP following tomentosin treatment. In HCT 116 cells, MMP was reduced by approximately 25% (*p* < 0.01), while in HT- 29 cells, a 20% reduction was observed (*p* < 0.0001), indicating that tomentosin induces mitochondrial dysfunction, which could lead to apoptotic cell death through the intrinsic pathway (Fig. 3A). ROS production was measured using a DCFDA assay, with increased fluorescence indicating higher ROS levels. The results showed that tomentosin significantly increased ROS production in colorectal cells. The baseline ROS levels in the control group were measured as 306.67 ± 15.69 RFU for HCT 116 cells and 366.33 ± 4.72 RFU for HT- 29 cells. Upon tomentosin treatment, ROS levels increased to 429 ± 16.09 RFU in HCT 116 and 462.33 ± 30.08 RFU in HT- 29, corresponding to 1.40-fold (*p* < 0.001) and 1.26-fold (*p* < 0.01) increases, respectively (Fig. 3B). These findings suggest that tomentosin induces oxidative stress in colorectal cancer cells, which could contribute to its anticancer activity by triggering and activating stress response pathways.

### Tomentosin enhanced the autophagic activity in colorectal cancer cells

**Fig. 4:**
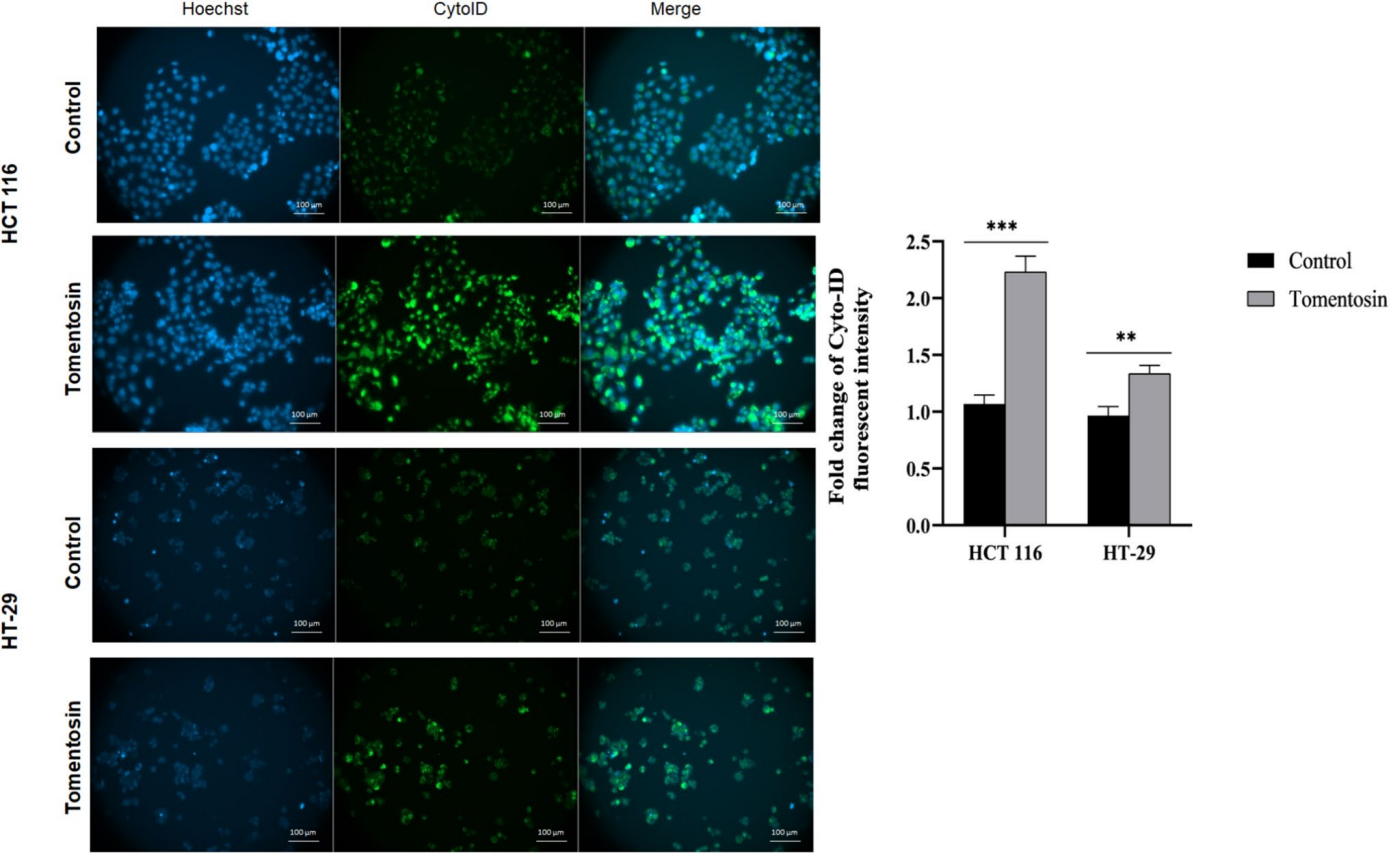
Tomentosin enhanced autophagic activity in colorectal cancer cells. Representative fluorescence microscopy images of HCT 116 and HT- 29 cells stained with Hoechst (blue) for nuclear visualization and Cyto-ID (green) to detect autophagic vesicles. The merged images highlight increased Cyto-ID fluorescence, indicating enhanced autophagic activity in tomentosin-treated cells compared to controls. Right panel shows the quantitative fold change in Cyto-ID fluorescence intensity in HCT 116 cells and HT- 29 (cells, representing the upregulation of autophagy. (**p < 0.01, ***p < 0.001)

Fluorescence microscopy images showed that Cyto-ID staining intensity (green) was notably higher in tomentosin-treated cells compared to the control group, indicating an increase autophagic activity. In addition, quantitative analysis of Cyto-ID fluorescence intensity, represented as fold change, demonstrated that tomentosin treatment resulted in a 2.3-fold increase in HCT 116 cells (*p* < 0.001) and 1.7-fold increase in HT- 29 cells (*p* < 0.01) (Fig. 4). These results suggest that tomentosin induces autophagy in colorectal cancer cells, contributing to its modulatory effects on cell death mechanisms.

#### Tomentosin modulated cell death and autophagy by regulating apoptotic, autophagic, and ER stress pathways

**Fig. 5:**
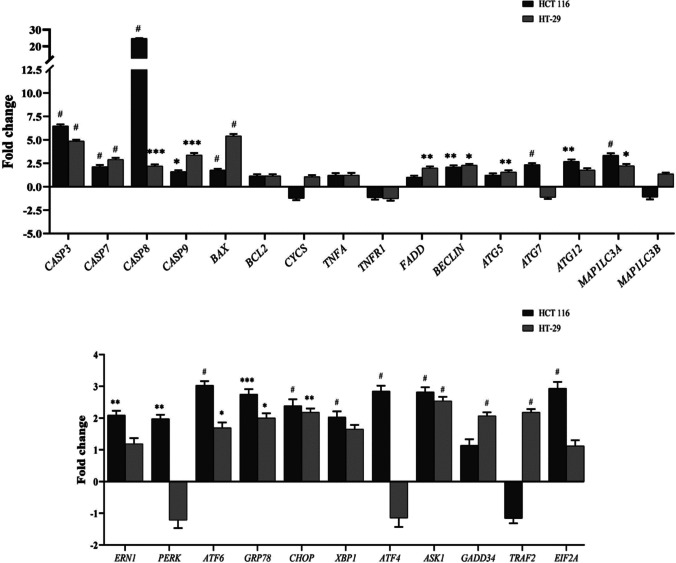
The mRNA expression levels of apoptosis-related (*CASP3, CASP7, CASP8, CASP9, BAX, BCL2, CYCS, TNFA, TNFR1, FADD*), autophagy-related (*BECLIN1, ATG5, ATG7, ATG12, MAP1LC3 A, MAP1LC3B*), and ER stress-related (*ERN1, PERK, ATF6, GRP78, CHOP, XBP1, ATF4, ASK1, GADD34, TRAF2, EIF2 A*) genes were evaluated using qRT-PCR in HCT 116 and HT- 29 cells after tomentosin treatment. Gene expression levels were normalized to GAPDH and presented as fold change relative to the control group (* *p* < 0.05, ** *p* < 0.01, *** *p * < 0.001***, # *p* < 0.0001)

The effects of tomentosin on the mRNA expression of key genes involved in apoptosis, autophagy, and ER stress were assessed using RT-qPCR. Tomentosin treatment significantly increased the expression levels of apoptosis-related genes in both HCT 116 and HT- 29 cells. In HCT 116 cells, the fold changes for *CASP3*, *CASP7*, *CASP8*, *CASP9*, and *BAX* were 6.5-fold (*p* = 0.000002), 2.13-fold (*p* = 0.00097), 24.72-fold (*p* = 0.000003), 1.61-fold (*p* = 0.041673), and 1.78-fold (*p* = 0.00005), respectively. In the HT- 29 cells, the corresponding fold increases were 4.87-fold (*p* = 0.000001), 2.9-fold (*p* = 0.00003), 2.2-fold (*p* = 0.0005), 3.4-fold (*p* = 0.0003), and 5.42-fold (*p* = 0.000008), respectively (Fig. 5). The upregulation of these genes in both cell lines confirm the induction of apoptosis via both intrinsic and extrinsic apoptotic pathways.

Tomentosin also significantly modulated autophagy-related genes. In HCT 116 and HT- 29 cells, *BECLIN* (2.11-fold, *p* = 0.003; 2.29-fold, *p* = 0.015, respectively) and *MAP1LC3 A* (3.35-fold, *p* = 0.00003; 2.23-fold, *p* = 0.04, respectively) were significantly upregulated, indicating autophagic processes. Additionally, *ATG5* (1.56-fold, *p* = 0.009) and *FADD* (1.99-fold, *p* = 0.006) showed significant upregulation only in HT- 29 cells, whereas *ATG7* (2.25-fold, *p* = 0.00005) and *ATG12* (2.7-fold, *p* = 0.005) were upregulated exclusively in HCT 116 cells (Fig. 5). Meanwhile, *BCL2*, *CYCS*, *TNFA*, and *TNFR1* did not exhibit significant expression changes (*p* > 0.05).

Furthermore, tomentosin induced ER stress-related genes, with *ATF6*, *GRP78*, *CHOP*, *XBP1* and *ASK1* significantly upregulated in both HCT 116 and HT- 29 cells. In HCT 116, the respective fold changes were 3.03-fold (*p* = 0.000001), 2.75-fold (*p* = 0.0001), 2.39-fold (*p* = 0.00002), 2.03-fold (*p* = 0.0004), and 2.85-fold (*p* = 0.00002), while in HT- 29, these genes were 1.69-fold (*p* = 0.03), twofold (*p* = 0.041), 2.18-fold, (*p* = 0.001), 1.65-fold (*p* = 0.006), and 2.54-fold (*p* = 0.00004), respectively. However, *ERN1* (2.09-fold, *p* = 0.002), *PERK* (1.98-fold, *p* = 0.007), *ATF4* (2.85-fold, *p* = 0.0000006), and *EIF2 A* (2.94-fold, *p* = 0.000026) were significantly upregulated only in HCT 116 cells (Fig. 5). These findings demonstrate that tomentosin triggers apoptosis and autophagy while also influencing ER stress pathways, contributing to its cytotoxic effects in colorectal cancer cells.

## Discussion

The present study demonstrated that tomentosin exerts significant anticancer effects in colorectal cancer cells by inhibiting cell viability, reducing clonogenic survival, suppressing invasion, inducing apoptosis, and modulating autophagic and ER stress pathways. Tomentosin treatment effectively disrupts multiple hallmarks of cancer, suggesting its potential as a promising anticancer agent. XTT assay results revealed that tomentosin significantly reduced the viability of HCT 116 and HT- 29 cells in a dose-dependent manner, with effective concentrations of 15 µM and 10 µM, respectively. Colony formation assays further confirmed the cytotoxic potential of tomentosin, as the number of colonies was markedly decreased in treated groups compared to controls. This suggests that tomentosin not only inhibits proliferation but also impairs the long-term survival of colorectal cancer cells.

Previous studies have reported the antiproliferative effects of tomentosin in various cancer types (Yu et al. 2021; Migheli et al. 2022; Güçlü et al. 2022). Tomentosin has been shown to inhibit proliferation in MG- 63 human osteosarcoma cells (Lee et al. 2019), AGS gastric cancer and MOLT- 4 leukemia cells (Yang et al. 2020; Yang et al. 2021), and SK- 28, 624 mel, 1363 mel melanoma cells (Rozenblat et al., 2008). Additionally, tomentosin suppressed HepG2 and Huh7 hepatocellular carcinoma cell proliferation in a dose- and time-dependent manner (Yu et al. 2021; Park 2019; See-Hyoung & Park 2019). Moreover, *Inula viscosa* leaf extracts were reported to inhibit colorectal cancer cell growth in vitro (HCT116, colo320) and in vivo (C57BL/6 mice transplanted with MC38 cells), promoting apoptosis (Bar-Shalom et al. 2019). Similarly, ethanolic extracts of *Inula viscosa* exhibited antiproliferative effects against HT- 29 cells and reduced oxidative stress in an in vivo colitis model (Kheyar et al. 2022). However, these studies did not specifically isolate tomentosin, making the present study the first to directly investigate its effects in colorectal cancer cells.

Mitochondrial dysfunction and oxidative stress are critical contributors to apoptosis. Our previous study demonstrated that tomentosin induces MMP loss in pancreatic cancer cells (Güçlü et al. 2022). In colorectal cancer cells, we present evidence that tomentosin triggers apoptosis and suppresses cell invasion, suggesting its potential role in suppressing metastasis. Our findings indicate that tomentosin induces MMP loss, suggesting disruption of mitochondrial outer membrane integrity and activation of apoptotic signals. The observed increase in the apoptosis index correlates with the upregulation of key apoptotic markers, including *CASP3, CASP7, CASP8, CASP9,* and *BAX*, indicating activation of both the intrinsic and extrinsic apoptotic pathways. Increased *BAX* levels and the lack of significant *BCL2* suppression suggest that mitochondrial permeability alterations may be directly linked to tomentosin-induced cellular stress. The intrinsic apoptotic pathway might be caused by more cytochrome C release and permeability of mitochondrial outer membrane brought on by the elevations in the ratio of BAX/BCL2. *Inula viscosa* extract supresses cell cycle and proliferation genes (*c-MYC, CCND1*) while inhibiting anti-apoptotic genes (*BCL2, BCL2L1, BCL11 A*), demonstrating significant anticancer effects on Burkitt’s lymphoma (BL) cells (Virdis et al. 2020). Tomentosin further inhibits survival pathways (PI3 K/AKT and JAK/STAT), inducing cell cycle arrest and apoptosis in BL cells. Tomentosin downregulates anti-apoptotic genes (*BCL2 A1, CDKN1 A*) and upregulates the pro-apoptotic gene *PMAIP1* (Virdis et al. 2021). Another study (Yu et al. 2021) proved that tomentosin lower viability and proliferation in HepG2 and Huh7 cells in a time- and dose-dependent manner. The same investigation reported *BCL- 2* downregulation alongside increased apoptotic markers, including phosphorylated *p53*, cleaved *PARP1*, and *FOXO3*, as well.

In this study, we found a notable increase in ROS levels following tomentosin treatment, aligning with Merghoub et al. (2017), who reported higher ROS and lower MMP in SiHa and HeLa cells. Similarly, Lee et al. (2019) showed that ROS generation facilitated tomentosin-induced apoptosis in MG- 63 osteosarcoma cells, with 40 µM tomentosin triggering G2/M arrest, reduced invasion, and upregulated *FOXO3* and *p27*. According to these results, it can be said that tomentosin induces mitochondrial dysfunction, with elevated ROS potentially leading to CRC cell death. Another study reported that tomentosin exhibited anti-inflammatory activity by inhibiting inflammatory mediators in non-cancerous RAW264 cells (Park et al. 2014). In addition, Yang et al. (2022) reported that tomentosin induced transient ROS production, leading to the activation of the NRF2 pathway through disruption of the NRF2–KEAP1 complex in human keratinocyte HaCaT cells (Yang et al. 2022). These findings indicate that tomentosin plays a role in regulating redox homeostasis in healthy cells, which may have implications for its broader biological activity.

High ROS levels can trigger the ER stress response by causing both DNA damage and disruptions in protein folding processes (Cao et al. 2014; Chen et al. 2023a). The present study results demonstrated that tomentosin treatment led to the upregulation of key ER stress markers, including *GRP78/BiP, ATF6, CHOP, XBP1,* and *ASK1,* indicating its critical role in ER stress responses. The induction of ER stress has been reported as a potential strategy for cancer therapy, as prolonged or unresolved ER stress can trigger apoptosis (Oakes 2020; Zhang et al. 2024). In this study, the increase of GRP78/BiP initially suggests that it may promote survival signals by regulating the unfolded protein response (UPR) of cells. However, the significant increase in CHOP expression in tomentosin-treated cells indicates that ER stress may contribute to apoptosis induction in colorectal cancer cells. Wang et al. (2019) reported that a sesquiterpene lactone, alantolactone, isolated from Inula helenium, induced ROS-mediated ER stress in lung cancer cells (A549, NCI-H520). Their findings demonstrated that increased ROS levels enhanced eIF2α phosphorylation and *CHOP* expression, both of which are key markers of ER stress-associated apoptosis (Wang et al. 2019). These results suggest that ROS-mediated ER stress plays a critical role in the cytotoxic effects of anticancer agents, which aligns with our findings on tomentosin-induced ER stress in colorectal cancer cells.

Beyond apoptosis, autophagic cell death is an another critical mechanism in the cancer progression (Coates et al. 2010). Through the lysosomal system, autophagy breaks down and recycles damaged organelles and proteins, enabling cells to endure stressful conditions. In addition, autophagy has a dual role. Excessively or dysregulated autophagy can result in increased cellular stress and cell death, whereas low autophagy levels sustain cellular homeostasis and encourage cancer cell survival (Galluzzi et al. 2018; Silva et al. 2020). In the present study, the increase in Cyto-ID fluorescence intensity and the upregulation of autophagy-related genes (*BECLIN1*, *MAP1LC3 A*, *ATG5*, *ATG7*, and *ATG12*) indicate that tomentosin significantly enhances autophagic activity. While excessive or uncontrolled autophagy can encourage cell death, autophagy can also serve as a pro-survival process. According to our study, autophagy may function as a protective mechanism in the early stages, allowing cells to adapt to tomentosin-induced stress. However, the increase in *BECLIN1* levels may stimulate autophagic processes, which are typically inhibited by *BCL2*. To further clarify the role of autophagy in tomentosin-induced cell death, future studies should incorporate autophagy inhibition assays using inhibitors such as chloroquine, hydroxychloroquine, or 3-MA to determine whether autophagy plays a cytoprotective or cytotoxic role in tomentosin-treated cells.

The precise function of autophagy in cancer remains unclear, though. Thus, more research is required to elucidate the effects of tomentosin administration on autophagic pathways. Tomentosin boosted autophagosome accumulation, elevated *LC3B-II* and *BECLIN1*, and lowered p62 levels, according to Wang et al. (2018), which supports our findings. Similarly, Chen et al. (2023b) found that by upregulating *LC3B* expression, isolinderalactone, another sesquiterpene lactone, triggered autophagy in CRC cells. Considering all these results, which validate our research, tomentosin not only triggers apoptosis but also regulates autophagy. Although our findings show that tomentosin has strong anticancer potential, more research is required to ascertain its pharmacokinetic characteristics, bioavailability, and in vivo effectiveness. Moreover, combination studies with standard chemotherapeutics could provide insights into its potential synergistic effects in colorectal and other cancer types. Additionally, pathway inhibition experiments, such as NAC for ROS inhibition, Z-VAD for apoptosis inhibition, 3-MA for autophagy inhibition, are needed to confirm causality. Finally, to further elucidate the broader cellular stress mechanisms associated with tomentosin treatment, future investigations should explore the NRF2, JNK, and p38 MAPK pathways.

## Conclusion

Natural compounds are extensively explored for their therapeutic potential in CRC due to their ability to target multiple pathways with minimal toxicity. Their accessibility, low toxicity, and multi-targeting capacity make them valuable in modern drug discovery. By triggering both intrinsic and extrinsic apoptotic pathways and modifying autophagy, the present study shows that tomentosin has anticancer effects on CRC cells. Treatment with tomentosin induces cellular stress and ultimately cell death through mechanisms such as mitochondrial dysfunction, ROS production, and ER stress response. These findings highlight tomentosin’s potential as a novel therapeutic agent for CRC, warranting further in vivo and clinical investigations.

## Data Availability

All source data for this work (or generated in this study) are available upon reasonable request.

## Declarations

### Constent to participate

Not applicable. This study does not involve human participants.
